# Establishing bioinformatics research in the Asia Pacific

**DOI:** 10.1186/1471-2105-7-S5-S1

**Published:** 2006-12-18

**Authors:** Shoba Ranganathan, Martti Tammi, Michael Gribskov, Tin Wee Tan

**Affiliations:** 1 Department of Chemistry and Biomolecular Sciences and Biotechnology Research Institute, Macquarie University, Sydney NSW 2109, Australia; 2 Department of Biochemistry, Yong Loo Lin School of Medicine, National University of Singapore, 8 Medical Drive, Singapore 117597, Singapore; 3 Department of Biological Sciences, National University of Singapore, 14 Science Drive 4, Singapore 117543, Singapore; 4 Department of Biological Sciences, Purdue University, Lilly Hall of Life Sciences 915 W. State Street, West Lafayette, IN47907-2054, USA

## Abstract

In 1998, the Asia Pacific Bioinformatics Network (APBioNet), Asia's oldest bioinformatics organisation was set up to champion the advancement of bioinformatics in the Asia Pacific. By 2002, APBioNet was able to gain sufficient critical mass to initiate the first International Conference on Bioinformatics (InCoB) bringing together scientists working in the field of bioinformatics in the region. This year, the InCoB2006 Conference was organized as the 5^th ^annual conference of the Asia-Pacific Bioinformatics Network, on Dec. 18–20, 2006 in New Delhi, India, following a series of successful events in Bangkok (Thailand), Penang (Malaysia), Auckland (New Zealand) and Busan (South Korea). This Introduction provides a brief overview of the peer-reviewed manuscripts accepted for publication in this Supplement. It exemplifies a typical snapshot of the growing research excellence in bioinformatics of the region as we embark on a trajectory of establishing a solid bioinformatics research culture in the Asia Pacific that is able to contribute fully to the global bioinformatics community.

## Introduction

The Asia-Pacific Bioinformatics Network (APBioNet, [1-3]) was established in 1998 [4] to bring together scientists from diverse disciplines, working together in the area of Bioinformatics to champion the advancement of bioinformatics in the Asia Pacific region. After annual meetings held at the Pacific Symposium of Biocomputing (1998–2001), APBioNet executive committee members assisted in organizing InCoB2002 (the International Conference on Bioinformatics, 2002) at Bangkok, Thailand and adopted this meeting as their annual conference. Subsequent conferences followed in Penang, Malaysia (2003); Auckland, New Zealand (2004) and Busan, South Korea (2005). InCoB 2006 was held New Delhi, India.

In the early years, we focused on constructing the network infrastructure capable of supporting the rapid dissemination of bioinformatics databases and computational resources throughout the region such as the BioMirrors initiative [5]. Education and training in bioinformatics in terms of awareness and advocacy amongst the life science community was a key priority, which led to initiatives such as the S* Life Science Informatics Alliance [6]. Today, we are starting to reap the fruits of our collective early labour. Conferences on bioinformatics ranging from the traditional Genome Informatics Workshop (GIW) [7] based in Japan to the Asia Pacific Bioinformatics Conference (APBC) and the International Life Science Grid Workshop (LSGRID) [8] are already showcasing the expertise of Asia Pacific bioinformatics research. In recognition of the tremendous growth of bioinformatics in this region, even the International Society for Computational Biology (ISCB) (MG is the current President), to which APBioNet is affiliated, chose to hold the annual flagship ISMB conference in this region in 2003 [9]. High quality research papers from the region have started to appear in bioinformatics publications originating in the region, such as the Journal of Bioinformatics and Computational Biology (World Scientific, Singapore) [10] and Applied Bioinformatics (originally from New Zealand) [11].

In recent years, bioinformatics research in the region has reached a standard, requiring international peer-reviewed high-impact factor journal publication. So in 2006, on the occasion of the 5^th ^Annual International Conference on Bioinformatics (InCoB) the APBioNet Executive Committee and the InCoB Steering Committee (chaired by one of the authors, SR) has embarked on establishing international standards in bioinformatics research through this vehicle of a special *BMC Bioinformatics *issue. Manuscripts were sought from APBioNet members in any area of bioinformatics. The submitted manuscripts span several active research areas, such as the development, organization, mining and integration of data resources; tools for the analysis of sequences, protein structure, transcriptomes, interactomes and genomes; immunoinformatics and the development of informatics resources for large-scale distributed grid computing.

### Proceedings summary

Papers submitted to these proceedings were peer-reviewed by at least two reviewers, from the APBioNet/InCoB editorial board members and external experts as required. The aim of the journal proceedings was to rigorously select only the top 22 high-quality papers originating from more than a dozen Asia Pacific countries, out of 48 manuscripts (acceptance rate of 46%) shortlisted from the 557 abstracts submitted to the scientific organising committee of InCoB 2006. The innovative bioinformatics research in the region is reflected in these accepted papers coauthored from Australia, China, India, Japan, Singapore, South Africa, Taiwan, The Netherlands and USA, which fall into several general themes as described in the following sections.

### Sequence analysis

In the realm of sequence comparisons, Foret *et al*. [12] have determined the optimal word size for exact and approximate matches between random sequences. Sprenger *et al*. [13] compare available subcellular localization prediction methods. Support vector machine (SVM) approaches have been applied to the prediction of caspase cleavage sites [14] and the functional class of metal-binding proteins [15].

### Structural bioinformatics

New methodologies for fast structure retrieval using spectral graph matching [16] and domain boundary prediction using a novel interdomain linker index [17] have been reported. A detailed modelling study of protein dimerization [18] describes how structural resources and methods can be applied to understand biologically functional quaternary structures.

### Transcriptome analysis

Transcriptome research is represented by four papers. The first two are novel approaches for predicting microRNA [19] and selecting effective siRNA [20] sequences. The application of probabilistic and SVM techniques to splice site identification [21] is followed by the promoter analysis of antimicrobial peptide-coding genes in three mammalian genomes [22].

### Interactomes

Protein-protein interactions and the networks they form are essential to the fundamental understanding of how biological pathways function. Interactome databases for yeast [23] and the fruit fly [24] are reported, to support organism-wide analyses. Mathivanan *et al*. [25] have critically evaluated resources available for the human interactome.

### Genome analysis

Curation and annotation of viral [26], bacterial [27] and zebrafish [28] genomes present interesting challenges, due to the range in genome size, variability and complexity. In the case of zebrafish, Christoffels *et al*. [28] have effectively used carp ESTs to facilitate the annotation.

### Text mining

Text mining approaches allow the integration of the scientific literature with genomic and high-throughput experiments.  Tsai *et al*. [29] present improvements in identifying biological terms, a key step in this process.

### Immunoinformatics

In the global fight against disease, Khan *et al*. [30] discuss sequence-based immunogenic epitope prediction strategies for the dengue virus, while Tong *et al*. [31] describe a structure-based docking approach to define HLA Class II autoantigenic peptides.

### Grid computing

As computational problems in biology evolve in size, resource requirements and complexity, grid computing has emerged as a viable solution in many instances. Konagaya [32] reviews currently available grid computing resources and applications, while providing a futuristic extension from a computing to a knowledge grid.

## Conclusion

This special issue of *BMC Bioinformatics* is a milestone in Asia Pacific bioinformatics research. The breadth and depth of research activity in the region, as reflected by the submitted papers, underscores the rapid development of bioinformatics amongst our institutions of higher learning and research. It demonstrates that the bioinformatics resource infrastructure set up in the past forms a strong foundation for meeting the demands of the research community. The success of regional bioinformatics education programs is highlighted by the increasing number of papers with graduate research students as first authors.

As bioinformatics starts to mature in the Asia Pacific region, research leaders, such as those in the recently formed Asian Association for Societies in Bioinformatics (AASBi) [33] and administrators of our respective institutions must plan for the network and resource infrastructure of the next decade, the so-called cyberinfrastructure for biology (CIBIO) [34]. Indeed, policy meetings such as the East Asia Bioinformatics Networking (EABN) [34], first held at InCoB2005 in Busan, and ASEAN human resource development in bioinformatics, held in India and China are already in place to address these issues. We are well placed "to foster the growth of Bioinformatics and its allied disciplines in the Asia Pacific" [1].  This growth is a key element in the development of a new generation of researchers who can lead in developing the new Biology of the twenty-first century.
